# In-Season Test–Retest Reliability of Visual Smooth-Pursuit (EyeGuide Focus) Baseline Assessment in Female and Male Field-Sport Athletes

**DOI:** 10.3390/jfmk9010046

**Published:** 2024-03-04

**Authors:** Ayrton Walshe, Ed Daly, Alan J. Pearce, Lisa Ryan

**Affiliations:** 1 Department of Sports, Exercise, and Nutrition, Atlantic Technological University, H91 T8NW Galway City, Ireland; ayrton.walshe@research.atu.ie (A.W.); ed.daly@atu.ie (E.D.); 2 School of Allied Health, Human Services and Sport, La Trobe University, Melbourne 3086, Australia

**Keywords:** eye tracking, sport-related concussion, saccades, ocular, athletes, mTBI

## Abstract

Sport-related concussions (SRCs) are a mild traumatic brain injury (mTBI) that induces transient symptoms in athletes. These symptoms provide avenues for developing emerging technologies to diagnose SRCs, in particular ocular and vestibular dysfunction. The following study aims to assess the reliability of visual smooth-pursuit technology (EyeGuide Focus) in amateur field-sport athletes. A convenience sample of 30 mixed-gender athletes (mean age = 24.89 ± 6.81 years) completed two testing sessions separated by 2–7 days. Participants were tested at rest, free from distraction, and completed a 10 s smooth pursuit while seated. Participants completed 2–4 practice trials before completing three tests at each session. Appropriate difference, reliability, and repeatability tests were performed in Statistical Packages for the Social Sciences. No significant difference existed between the time points (*p* > 0.05). The reliability between sessions was poor (ICC = 0.24; 95% CI = 0.03–0.42), and the mean coefficients of variation were 20% and 21% for each session, indicating poor repeatability. However, the implementation of practice trials did prevent the familiarization effects that are evident in the previous literature (*p* > 0.05). The within-session reliability of EyeGuide Focus has varied from poor (ICC ≤ 0.50) to good (ICC = 0.75–0.90) in the previous literature, indicating the fact that greater research is required before this tool can be implemented in applied settings.

## 1. Introduction

Sport-related concussions (SRCs) are defined as a mild traumatic brain injury (mTBI) whereby force is applied to the head (or transmitted upward to the head), which induces a metabolic cascade within the brain and causes a varying range of transient symptoms in athletes [1]. The injury can provide acute burden (0–14 days), but this may extend into months in post-concussive syndrome [2]. The long-term consequences of repeated head impacts are also becoming apparent in athletes from multiple sporting backgrounds [3,4].

Competition SRC rates in female rugby union range from 8.20 to 16.11 per 1000 h, and 4.04 per 1000 athletic exposures in association football [5]. In male sport, match rates of 12.00 per 1000 h were found in elite rugby union [6], and 1.77 per 1000 athletic exposures in male association football [7]. There is evidence to support a disparity in SRC rates between male and female athletes; however, many female sports have minimal and severe variance in injury surveillance and healthcare provision at amateur and elite levels [5,8,9]. Additionally, both male and female athletes have been seen to have poor attitudes toward SRC disclosure and return to play adherence [9,10,11]. As a result, clinical tools that can identify SRCs without symptom disclosure are imperative to athlete welfare.

Clinically speaking, SRC diagnosis and prognosis are not straightforward processes. To be more precise, the reliability and repeatability of current SRC diagnosis methodologies are often sub-optimal with varying levels of success in achieving desirable reliability [12]. Research has also identified that variables such as exertion [13,14], learning effects [15], or attention deficit hyperactivity disorder (ADHD) [16] can have confounding effects on such tools. For these reasons, it is currently advised that a multi-modal assessment such as the Concussion in Sport Group’s (CISGs) Sport Concussion Assessment Tool (SCAT) [17] be used to better detect SRCs [18]. However, each of these methodologies requires medical professionals to administer, which can be time-intensive for those not specialized in sports medicine [19]. This means many athletes in community sports may not be provided an opportunity for such assessments due to a lack of medical coverage, finances, resources, or poor attitudes toward them [9].

The most recent CISG consensus statement on SRCs has acknowledged the benefit of emerging technologies in concussion research, with an understanding that greater research is needed before such tools are implemented [1]. One tool in particular, EyeGuide Focus, offers a 10 s digital assessment of visual smooth pursuits, which is commonly incorporated into vestibular–ocular motor screening (VOMS) [20,21]. Visual smooth pursuits can be impaired in 43–60% of patients who experience an mTBI and are therefore a target during assessment and rehabilitation of dysfunction post-injury [22,23]. Technological tools like EyeGuide Focus, which evaluate smooth pursuits, may require less clinical expertise to operate than established tests. In theory, with their time-efficient protocols, they would prove invaluable in a community sports setting to help support coaches, volunteers, medical professionals, and allied healthcare professionals in their decision-making processes.

EyeGuide Focus has achieved varying levels of intra-session reliability in recent research, and learning effects have been identified in athletes [24,25]. To the authors best knowledge, no study of inter-session reliability has been conducted with the tool to date. The device has also been used beyond SRC research in peer- and non-peer-reviewed studies to explore workplace fatigue [26], the effectiveness of visuomotor training [27], and the impact of mixed martial arts bouts on scoring [28]. Age-related differences in EyeGuide Focus scoring between adults and adolescents have been observed [24]. Such differences in ocular assessments of young athletes are not uncommon and emphasize the need for periodical baseline assessments [29,30]. It is currently unclear if further differences in EyeGuide Focus scoring exist between adults of different age groups at this time.

Given the potential for benefit and acceptability of this tool in community sport, the following study aims to determine the test–retest reliability of EyeGuide Focus in male and female field-sport athletes. This study also aimed to determine if practice trials can offset familiarization effects with the device and if factors such as sleep quality, SRC history, and age may affect EyeGuide Focus scoring.

## 2. Materials and Methods

This manuscript has been produced in accordance with the Guidelines for Reporting Reliability and Agreement Studies (GRRAS) and is available in Supplementary File S1 [31]. An a priori sample size was calculated in G-Power using pre-set values of α (0.05), power (1 − β = 0.95), and effect size (0.25), requiring a sample size of 44 participants. The study was advertised within ATU Galway, on social media, and by contact with amateur sports clubs near the university. A sample of 43 participants originally agreed to participate in the study (female = 59.50%). To be included, participants had to be healthy, aged 18 or older, and currently participating in one or more contact or collision sports. Participants were excluded if they currently had an SRC, if they had a history of five or more SRCs (*n* = 3), if they had ADHD or similar, if consent forms were not returned (*n* = 1), if they failed to attend both testing sessions (*n* = 6), if a baseline score could not be obtained on the testing units (*n* = 2), or if data were lost due to device malfunction (*n* = 1). As such, following the application of the exclusion criteria, 30 athletes were included in the final analysis. Participants were primarily involved in rugby union (*n* = 11), soccer (*n* = 6), Gaelic football (*n* = 10), camogie (*n* = 2), and field hockey (*n* = 1).

Interested participants completed online informed consent and demographic questionnaires on Microsoft Forms before being timetabled for an assessment. These assessments primarily took place either two or seven days apart, depending on the subject’s availability and training schedule. A 48-h window was preferred, but to prevent dropouts, retesting was performed at the participants discretion. The demographic intake questionnaire included informed consent, age, sex, sport participation, SRC history data, confirmation of ADHD or similar, non-limiting visual impairment (e.g., myopia, astigmatism), and medication usage. The questionnaire also included a Groningen Sleep Quality Scale (GSQS) questionnaire, which assessed the previous night’s sleep via 15 true or false statements [32,33]. This GSQS was also repeated on day two. GSQS scores range from 0 to 14, with scores of 0–2 indicating normal sleep and scores ≥ 6 indicating disturbed sleep. To provide greater detail in this study, scores of 3–5 were deemed intermediate disturbed sleep.

**Figure 1:**
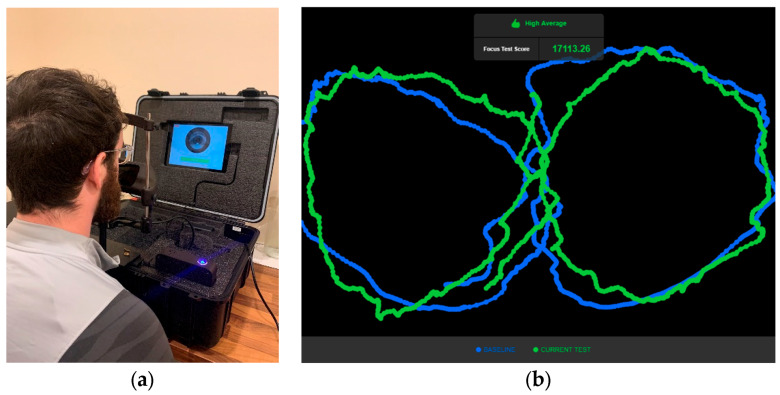
(**a**) EyeGuide unit set up, and (**b**) sample results output from the device.

EyeGuide Focus (EyeGuide, Lubbock, TX, USA) operates as an encased unit (Figure 1) consisting of an infrared camera built into a head rest unit and an iPad with the EyeGuide application installed. Upon opening, the head rest unit is pinned onto the front of the unit, and both the heights of the chin/head rest can be adjusted to suit each participant. The camera is then plugged into an external power source for operation. During assessment, participants track a white dot on a black background, performing an anti-clockwise ‘lazy 8’ pattern for 10 s. The unit camera then records the eye positioning at a frequency of 60 Hz, with the first and last seconds of the trace trimmed to reduce primacy effects [21]. Upon test conclusion, a confirmation of a valid or invalid test will be displayed. This will then be followed by an image of the participant’s trace, along with their score in arbitrary units (AUs) and their grade (“Superior”, “High Average”, “Above Average”, “Average”, “Low Average”, “Impaired”, and “Severely Impaired”). These scores were therefore not blinded to the participant or investigator. The scores were automatically calculated using the distance between the stimulus data and the position of the participant’s pupil; lower scores were indicative of a better performance on the test. Unsuccessful scores were indicated by participants scoring one standard deviation (SD) above or below both their baseline and EyeGuide’s demographic mean score.

Participants were assessed in a medical room at ATU Galway or off-site at their local club grounds. The room was prepared to be dark, quiet, and free from distraction. A number of steps were taken to ensure that this environment was maintained. Curtains were drawn, and a black-paned divider (VEVOR 6 Panel Room Divider, Taicang Vevor Machinery Equipment, Shanghai, China) was used where needed to further darken the testing area. This was essential for optimal use of the infrared camera, while noise-cancelling headphones (Bose Quiet Comfort 45, Bose Corporation, Framington, MA, USA) were placed on the participant during testing. Participants also placed a heart rate monitor (Polar Unite, Polar Electro, Kempele, Finland) upon entering the room to confirm and record a resting heart rate.

Participants would be seated before placing their chins in the EyeGuide unit. The unit camera and chin rest would then be adjusted to ensure the participants’ line of sight had a 0° angle of approach. The participant would be correctly seated if their pupil was detected at all times by the camera AI crosshair as they looked at each corner of the encased iPad. The participant would also place their hands on the desk or unit during testing to prevent fidgeting or excess movement during testing. During each test, the investigator would stand in front of the participant on the left side of the table to observe their performance. This aided in detecting blinking or head movement during tests and allowed investigators to determine if poor or invalid scores were caused by poor technique.

Participants performed 2–4 practice trials before performing three official trials at each testing session. These were performed as familiarization effects have been detected in previous research with the device [24]. Participants were given 30–60 s between subsequent measurements to prevent fatigue. A longer duration was not selected as functionality issues (i.e., lagging and force closing) were present in the device. Thus once operational, tests were conducted before issues could reoccur. If a baseline score could not be determined after five attempts, the participant was excluded from analysis.

Testing was conducted by one investigator (AW) and two department interns. The lead investigator (AW) completed training on the device with the license company and had remote access to staff for technical assistance where needed. AW completed individual training with both interns, completed mock testing with each, and supervised their first two assessments to ensure the protocol was stringently adhered to. AW is currently undertaking a PhD exploring SRCs in female athletes. The interns were enrolled in biomedical and medical-related degrees in Ireland and the USA during data collection.

All data were exported to an Excel spreadsheet (Microsoft, Redmond, WA, USA) and anonymized. Assumptions of normality were met (W = 0.99, *p* = 0.19); thus, parametric tests were performed. Paired sample *t*-tests and independent *t*-tests investigated if differences existed between subgroups, and one-way analysis of variance (ANOVA) tests investigated differences between trials. A two-way mixed-methods absolute agreement intra-class correlation coefficient (ICC) was used to assess the reliability of scores. ICC scores were classified as follows: ≤0.50 = poor; >0.50–<0.75 = moderate; ≥0.75–≤0.90 = good; and >0.90 = excellent reliability [34]. Two measures of effect sizes were used [35]. Hopkins interpretation of Cohens effect sizes (d) were classified as ≤0.20: trivial; >0.20–0.60 = small; >0.60–1.20 = moderate; >1.20–2.00 = large; and >2.00–4.00 = very large [36,37]. Eta squared effect sizes (*η^2^*) were classified as ≥0.01–<0.06 = small; ≥0.06–<0.14 = moderate; and ≥0.14 = large [38].

## 3. Results

### 3.1. Summary Analysis

In total, six scores were obtained from 30 participants (*n* = 180) who completed three official tests at both testing sessions. The participants’ mean age was 24.89 ± 6.81, and these were grouped as those aged 18–24 and those aged > 25 years to investigate if age-related differences were present in adults. The sample included 17 females and 13 males, and almost half of the participants had previously received an SRC (*n* = 14, median = 1 SRC). A total of 21 SRCs were disclosed and were primarily professionally diagnosed (*n* = 15) rather than self-diagnosed (*n* = 6). No participant had previously tested on the EyeGuide Focus unit, nor had ADHD or similar conditions. Three participants were currently taking birth control, and one participant was taking spironolactone. Six participants reported a visual impairment of some level (myopia, astigmatism, etc.), but this did not affect their sports participation. Mean GSQS scores were 4.40 ± 3.77 for day one (normal: *n* = 13; intermediate: *n* = 6; disturbed: *n* = 11) and 4.36 ± 3.66 (normal: *n* = 13; intermediate: *n* = 6; disturbed: *n* = 9; not provided: *n* = 2) for day two (*p* = 0.61, d = 0.10). The mean resting HRs were 69.16 ± 7.63 and 68.87 ± 8.26 beats per minute (*p* = 0.84, d = 0.04).

**Figure 2:**
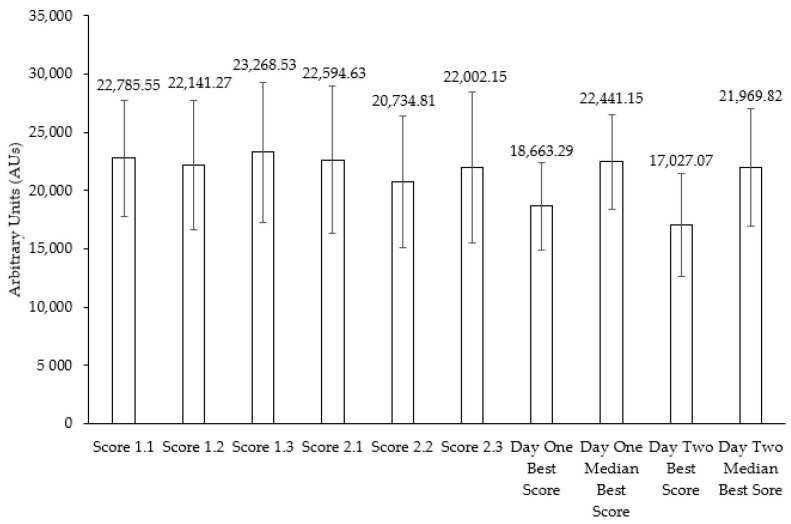
Group mean EyeGuide Focus scores obtained on days one and two. No significant difference was present between time points, best, and median best scores (*p* > 0.05).

The mean EyeGuide Focus scores for days one and two were 22,731.78 ± 5542.67 and 21,777.20 ± 6214.84 Aus (see Figure 2). A one-way ANOVA identified no significant difference and a small effect between time points (F(5,174) = 0.645, *p* = 0.665, η^2^ = 0.018). Paired sample *t*-tests identified no significant difference for median best (t(29) = 0.477, *p* = 0.637, d = 0.087) and best score (t(29) = 1.802, *p* = 0.082, d = 0.329) between days one and two. These effect sizes were deemed small for the best score but trivial for the median best score.

Independent samples *t*-test found that significant differences were present between previous and non-previously concussed individuals at score 1.3 (t(28) = −2.426, *p* = 0.01, d = −0.89), score 2.2 (t(28) = −2.889, *p* = 0.004, d = −1.06), and median best score (t(28) = −2.754, *p* = 0.010, d = 1.008), representing moderate effect sizes present for each. Significant differences were also found for visual impairment at score 1.2 (t(28) = −2.078, *p* = 0.047, d = −0.897) and for age group at score 1.1 (t(28) = −2.159, *p* = 0.040, d = 0.805). No other significant difference was found across tests 1.1–2.3, nor across the best and median best scores for previous SRC, visual impairment, or age group (*p* > 0.05). No significant difference was found for sex, sport, medication usage, or GSQS score across all tests (*p* > 0.05).

### 3.2. Reliability and Repeatabiliy Analysis

Test–retest reliability scores between trials are available in Table 1. Negative lower-bound confidence intervals for ICCs were present. However, true ICC cannot equal < 0, and thus, the ICC lower-bound confidence intervals were adjusted to equal 0 in such cases.

**Figure 3:**
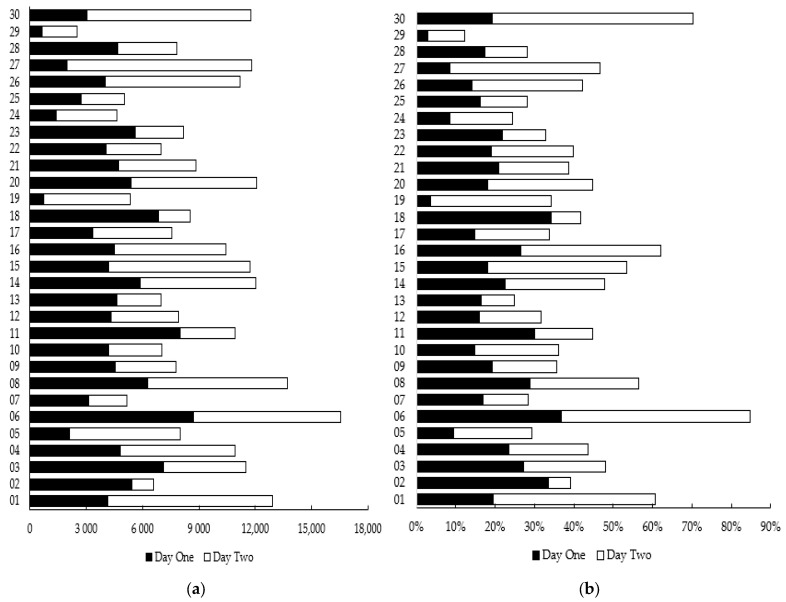
(**a**) Within-subject standard deviations and (**b**) coefficient of variations (CVs) for days one and two highlight large variability within and across participants.

ICC classifications between individual tests and testing days were either poor (≤0.50) or poor to moderate (≤0.50 to <0.75). Overall, ICCs were 0.27 (95% CI = 0.04–0.51) and 0.29 (95% CI = 0.07–0.53) for days one and two, respectively; mean CVs were 20% and 21%; and mean within-subject standard deviations were 4374.43 and 4706.27 AUs. A breakdown of within-subject variability across each testing day for each participant is available in Figure 3 and highlights these findings in detail.

**Figure 4:**
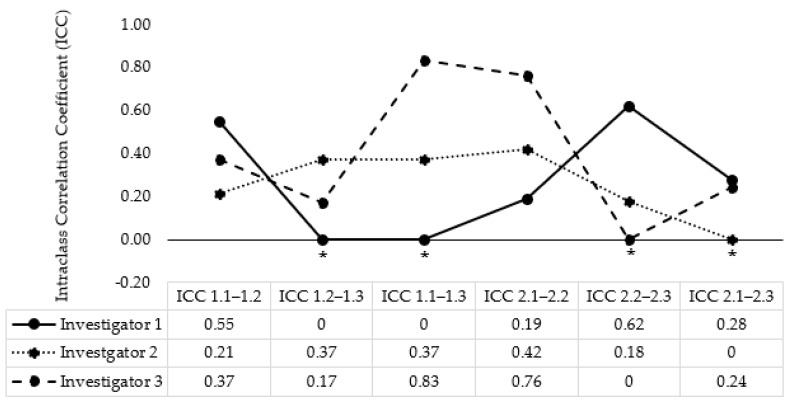
Intraclass correlation coefficients (ICCS) across investigator 1 (*n* = 12), investigator 2 (*n* = 7), and investigator 3 (*n* = 4). * indicates that the ICC estimate was negative and adjusted to 0.00.

Of the total test–retest participants (*n* = 30), most were conducted by the same investigator at each time point (*n* = 23; tester 1; *n* = 12, tester 2; *n* = 7, tester 3; *n* = 4). ICCs for each investigator are available in Figure 4. Test–retest reliability between days was 0.04 (95% CI = 0.00–0.36), 0.22 (95% CI = 0.00–0.59), and 0.20 (95% CI = 0.00–0.66) for testers one, two, and three, respectively.

## 4. Discussion

The present study assessed the inter- and intra-session reliability of EyeGuide Focus in female and male field-sport athletes. While statistical comparisons found no significant differences in the overall scores between sessions, the tool was found to have poor or poor-to-moderate reliability between subsequent trials and days. Reliability did not improve with the utilization of a best or median best score metric.

To our knowledge, this is the first study to successfully implement pre-test familiarization using EyeGuide Focus (see Figure 2). These findings have shown that 2–4 practice trials are sufficient to offset familiarization effects seen in previous EyeGuide Focus research [24]. The group scores obtained in the current study (mean = 22,254.49 ± 5907.67 AUs) are lower than those reported in the original EyeGuide study (mean = 29,633.05 ± 9209.84 AUs) [21], higher than those reported in pro mixed-martial arts athletes (mean = 17,426.06 AUs) [28], but almost identical to scores recently reported in adults in their first trial (mean = 22,503.33 ± 7014.32) [24].

The findings indicate that the reliability of EyeGuide Focus (see Table 1) is lower than comparable visual assessment tools such as the King-Devick (ICC = 0.89) and VOMS (ICC = 0.76; 96% CI = 0.64–0.84) [15,39]. However, it is interesting to note that Kontos et al. (2020) reported smooth pursuits obtained the lowest reliability of all seven VOMS components (ICC = 0.60; 95% CI = 0.42–0.73). The current study was the first to assess the inter-session reliability of EyeGuide Focus. Two studies have previously assessed the same-day reliability and repeatability of three repeated trials with conflicting findings. Pearce et al. (2023) recently found good overall intra-session reliability (ICC = 0.79; 95% CI = 0.70–0.86) in the tool with a mixed age (adolescent/adult) and sex population. Meanwhile, Fuller and Brown found poor overall reliability (ICC = 0.48; *Log transformed) in elite male rugby union athletes [25]. The current study found lower intra (day one ICC = 0.27; 95% CI = 0.04–0.51), day two ICC = 0.290; 95% CI = 0.07–0.53), and inter-session (ICC = 0.24; 95% CI = 0.03–0.42) reliability than these studies but achieved similar ICC classification to Fuller and Brown.

There may be a number of reasons for the variations observed with this tool to date. Pearce et al. recruited a population of physically active participants with no history of concussion, TBI, or visual conditions who were also not taking medication [24]. This differs from the present study and Fuller and Brown’s study, where previously concussed athletes and those with visual conditions were not excluded [25]. Such athletes were not excluded in the present study, as visual conditions are common in the general population [40,41], and this study aimed to assess the reliability of this tool for community sports settings. This study also aimed to assess the reliability of the tool in the context of sports where there are documented SRC rates and where the tool is likely to be used. Thus, including previously concussed athletes (who may be at higher risk for subsequent SRCs) was essential in the context of assessing the utility of this tool [42].

Indeed, some 95% ICCs presented in this study required adjusting to 0.00. Negative ICCs are theoretically impossible, and were possibly impacted by the variability in the dataset, the relatively small sample size, or the true ICC being close to 0.00 [43,44]. This variability is evident in the within-subject SDs and coefficients of variation presented for each participant in Figure 3, which further highlights the variability within and between participants across each testing session using the device. The findings of intra-rater reliability indicative of differences in ICCs between testers presented in Figure 4 are likely impacted by the sample size of each tester, with the smaller samples reducing the bandwidth for variability.

There are a number of avenues for future research from the present study. Ultimately, it is clear that EyeGuide Focus is not viable for implementation in its current format or without further investigation. It should also be stated that EyeGuide Focus is not intended to be used in lieu of a clinical diagnosis for SRCs, and clinical decisions lie with medical professionals as recommended by CISG [1] and indeed the manufacturers. These technologies should be regularly updated and modified as research and data collection expand with the device in question; as such, the model and software version in question become highly relevant and must be reported to accurately compare studies.

Given the variability within the tool, a practical recommendation of this study is the establishment of a controlled environment that can be replicated in future EyeGuide Focus research. Building on this point, given the poor reliability of the present study and the presence of some differences between subgroups, subsequent research may control for factors such as SRC history and visual impairment, as achieved by Pearce et al., before expanding to these populations [24]. It may also be useful to assess pre-test screen time, as this was mentioned by some participants during their assessment. Lastly, future research may also investigate the effect of exercise on the tool’s accuracy to assess whether the tool can be utilized in pitch-side assessments of athletes. This is warranted given the evidence that exercise affected some, but not all, ocular assessments for SRCs [14,45].

This study does have a number of limitations, however, in particular, this reliability study took place intra-season for each of the sports included. It was not possible to circumvent this issue due to logistical and research timeline restrictions, although our findings still meet the ICC classifications of Fuller and Brown, who conducted their testing during the pre-season [25]. This study also failed to recruit a statistically significant sample. Technical issues were present within the EyeGuide Focus unit, which sometimes caused lagging, forced closing, and the loss of participant data (*n* = 1). This led to additional trials for abnormal scoring and meant that the rest duration between trials was short to complete tests while the unit was operational. There were also participants recruited (*n* = 2) who could not obtain a baseline score on the EyeGuide Focus unit, an issue that has been reported in previous research [24]. It is unclear why some participants cannot achieve a baseline score on the unit, and this phenomenon requires further investigation.

## 5. Conclusions

EyeGuide Focus achieved poor reliability and repeatability between subsequent trials and days. This study has indicated that emerging technologies such as EyeGuide Focus require further development and research before being implemented in community sports. The study did find that 2–4 practice trials were sufficient to offset learning effects with the unit and should be implemented in future EyeGuide Focus research.

## Figures and Tables

**Table 1:** Intra-class correlation coefficients (ICCs), 95% confidence intervals (CIs), and classifications provided by Koo and Li. * indicates that the ICC estimate was negative and adjusted to 0.00.

Test	ICC	ICC (95% CI)	Classification
Score 1.1–1.2	0.39	0.04–0.66	Poor to Moderate
Score 1.2–1.3	0.18	* 0.00–0.50	Poor
Score 1.1–1.3	0.25	* 0.00–0.56	Poor to Moderate
Score 2.1–2.2	0.49	0.18–0.72	Poor to Moderate
Score 2.2–2.3	0.33	* 0.00–0.61	Poor to Moderate
Score 2.1–2.3	0.07	* 0.00–0.42	Poor
Day 1 and 2 (raw scores)	0.24	0.03–0.42	Poor
Day 1 and 2 (median best score)	0.34	* 0.00–0.62	Poor to Moderate
Day 1 and 2 (best score)	0.28	* 0.00–0.57	Poor to Moderate

## Data Availability

Data may be made available upon reasonable request.

## Supplementary Materials

The following supporting information can be downloaded at: https://www.mdpi.com/article/10.3390/jfmk9010046/s1. Supplementary File S1. GRRAS checklist for reporting of studies of reliability and agreement.
