# Supplementary material for: In-Season Test–Retest Reliability of Visual Smooth-Pursuit (EyeGuide Focus) Baseline Assessment in Female and Male Field-Sport Athletes
Source: J Funct Morphol Kinesiol. 2024 Mar 4;9(1):46. doi: 10.3390/jfmk9010046 (PMC10971711; doi:10.3390/jfmk9010046)
Supplement: Supplementary file 1 [file jfmk-09-00046-s001.zip › jfmk-2874422-supplementary.pdf]

GRRAS checklist for reporting of studies of reliability and agreement

Version based on Table I in: Kottner, J.; Audigé, L.; Brorson, S.; Donner, A.; Gajeweski, B.J.; Hróbjartsson, A.; Roberststs, C.; Shoukri, M.; Streiner, D.L. Guidelines for reporting reliability and agreement studies (GRRAS) were proposed. J. *Clin. Epidemiol.* **2011**, *64*, 96–106.

**Section**	**Item #**	**Checklist Item**	**Reported on Page #**
Title/Abstract	1	Identify in title or abstract that interrater/intrarater reliability or agreement was investigated.	#Page 1, Line 2.
Introduction	2	Name and describe the diagnostic or measurement device of interest explicitly.	#Page 2, Line 60-74
	3	Specify the subject population of interest.	#Page 2, Line 76-77
	4	Specify the rater population of interest (if applicable).	N/A
	5	Describe what is already known about reliability and agreement and provide a rationale for the study (if applicable).	#Page 2, Line 66-74
Methods	6	Explain how the sample size was chosen. State the determined number of raters, subjects/objects, and replicate observations.	#Page 3, Line 84-96<br>#Page 4, Line 147-148
	7	Describe the sampling method.	#Page 3, Line 85-87
	8	Describe the measurement/rating process (e.g. time interval between repeated measurements, availability of clinical information, blinding).	#Page 3, Line 109 - #Page 4, Line 128-158
	9	State whether measurements/ratings were conducted independently.	#Page 4, Line 154-158
	10	Describe the statistical analysis.	#Page 5, Line 160-171
Results	11	State the actual number of raters and subjects/objects which were included and the number of replicate observations which were conducted.	#Page 5, Line 174-175
	12	Describe the sample characteristics of raters and subjects (e.g. training, experience).	Subjects: #Page 5, Line 174-187<br><br>Raters: #Page 4, Line 158-160.
	13	Report estimates of reliability and agreement including measures of statistical uncertainty.	#Page 6, Table 1.<br>#Page 7, Figure 3<br>#Page 8, Figure 4
Discussion	14	Discuss the practical relevance of results.	#Page 8, Line 236-248
Auxiliary material	15	Provide detailed results if possible (e.g. online).	
